# Control Analysis of Cooperativity and Complementarity in Metabolic Regulations: The Case of NADPH Homeostasis

**DOI:** 10.3390/metabo13040485

**Published:** 2023-03-28

**Authors:** Benjamin Pfeuty, Julien Hurbain, Quentin Thommen

**Affiliations:** 1 Univ. Lille, CNRS, UMR 8523-PhLAM-Physique des Lasers Atomes et Molécules, F-59000 Lille, France; 2 Univ. Lille, CNRS, Inserm, CHU Lille, Institut Pasteur de Lille, UMR9020-U1277-CANTHER-Cancer Heterogeneity Plasticity and Resistance to Therapies, F-59000 Lille, France; quentin.thommen@univ-lille.fr

**Keywords:** regulation, metabolism, control analysis, kinetic model

## Abstract

Complex feedback regulation patterns shape the cellular metabolic response to external or internal perturbations. We propose here a framework consisting of a sampling-based metabolic control analysis of kinetic models to investigate the modes of regulatory interplay in metabolic functions. NADPH homeostasis, for instance in a context of oxidative stress, is an example of metabolic function that involves multiple feedback regulations which raises the issue of their concerted action. Our computational framework allows us to characterize both respective and combined effects of regulations, distinguishing between synergistic versus complementary modes of regulatory crosstalk. Synergistic regulation of G6PD enzymes and PGI enzymes is mediated by congruent effects between concentration sensitivities and reaction elasticities. Complementary regulation of pentose phosphate pathway and lower glycolysis relates to metabolic state-dependent range of regulation efficiency. These cooperative effects are shown to significantly improve metabolic flux response to support NADPH homeostasis, providing a rationale for the complex feedback regulation pattern at work.

## 1. Introduction

Metabolic control analysis (MCA) provides a rigorous theoretical framework to study the sensitivity of metabolic networks with respect to biochemical and environmental variations [1,2]. MCA has been further developed and expanded in several directions related to regulation, thermodynamics, or statistical analysis [3,4,5,6,7,8,9]. These developments contribute to more comprehensive analysis of control properties of metabolic networks with the challenging goal to decipher the logic of complex regulation pattern, such as those involving direct metabolite–enzyme interactions and coupling distal parts of a network [10,11,12].

The role of complex feedback regulatory scheme in shaping the network response to environmental changes is recognized in many contexts ranging from nutrient utilization, end-products homeostasis, or stress response [13,14,15]. This is illustrated by the cellular function of NADPH homeostasis, which involves the concerted action of a broad set of metabolic regulation [16,17,18]. NADP(H) homeostasis, like NAD(H) homeostasis [19], is important to keep a functional redox balance against diverse perturbations due for instance to oxidative stress, metabolic stress [20], or reductive biosynthesis [21]. Among the few metabolic pathways producing NADPH [22,23], the oxidative branch of the pentose phosphate pathway (oxPPP) is the main source of NADPH and is also stringently regulated by a set of allosteric and oxidative regulations [16]. Since the characterization of the feedback inhibition of G6PD and 6PGD through competitive binding of NADPH [24,25,26], the metabolic regulatory picture has became increasingly refined with evidences of a significant role for 6PG-dependent inhibition of PGI [17,27] or for oxidative inhibition of several glycolytic enzymes such as GAPD, PFKFB3, or PKM2 [28,29,30,31]. The regulation of NADPH homeostasis is therefore a valuable case study to assess methods and framework to investigate complex regulation patterns.

To address systemic properties of regulation patterns, our kinetic modeling framework extrapolates metabolic control analysis beyond a reference state, by combining mathematical analysis of control equations and sampling analysis of kinetic space. The idea is to represent the statistical distribution of control coefficients on low-dimensional subspaces defined and constrained by control equations. We first depict a global picture of the control pattern related to NADPH homeostasis driven by oxPPP in the absence of regulation, which reveals some trends which are in contradiction with experimental evidences. Combined mathematical and sampling analysis of control pattern further reveals how the presence of feedback regulation promotes PPP flux rerouting and NADPH homeostasis, involving also synergistic and complementary modes of cooperation. NADPH-dependent inhibition of G6PD and 6PG-dependent inhibition of PGI exhibits synergistic cooperation due to congruent concentration control of 6PG and G6P metabolites. Such a regulatory scheme must be supplemented with feedback inhibition of lower glycolysis to extend the efficiency range of NADPH homeostasis beyond a reference flux state.

## 2. Materials and Methods

**Figure 1:**
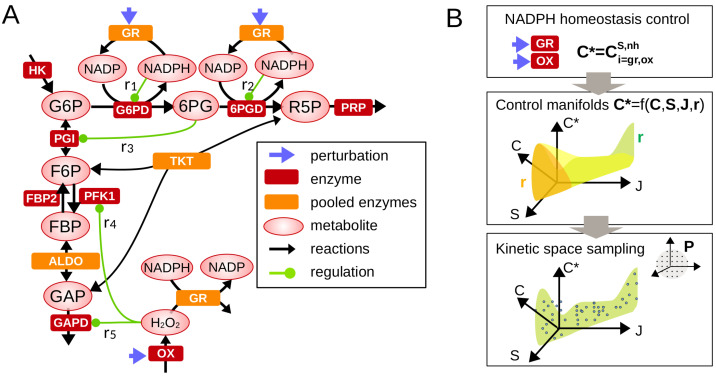
Control analysis of metabolic regulation involved in NADPH homeostasis. (**A**) A simplified metabolic network comprising the upper glycolysis and pentose phosphate pathways which includes a selected set of feedback regulation $r_{i}$ contributing to NADPH homeostasis. Legend is shown in inset. (**B**) Framework combining metabolic control analysis (Equations (3) and (4)) and sampling analysis of regulatory crosstalk.

To investigate metabolic control properties associated with feedback regulations, we consider the following kinetic modeling framework restricted to the main pathways involved in PPP-driven maintenance of NADPH homeostasis (Figure 1A). The concentration and flux dynamics of metabolic networks is commonly described by an ordinary differential equation system (see Appendix A),

(1) $$\frac{ds}{dt}=N\;v\left(s,p,r,t\right),$$

where vectors and matrices are denoted with bold and italic font styles, respectively. *N* defines the stochiometric matrix, $s_{i=1,n_{m}}$ the concentration of metabolite species, and $v_{i=1,n_{r}}$ the reaction rate functions described by mass-action kinetics involving rate constants $k_{i}$ and equilibrium constants $K_{i}$ gathered in $p=\{k_{i},K_{j}\}$ while regulatory parameters $r_{i}$ are treated separately. Steady-state concentrations S satisfy:

(2) Nv(S(p,r),p,r)=0,

and the associated steady-state flux vector is noted J(p)=v(S(p),p), where capitalized letters indicate steady-state quantities. Implicit differentiation of the steady-state Equation (2) with respect to kinetic parameters establishes a matrix expression for control coefficients and elasticities [32]:

(3) $$C^{J}=I+\epsilon \left(J,S,r\right)\;C^{S},$$

where the control and elasticity matrix coefficients are given by $C_{i}^{X,j}=\frac{\partial \ln X_{j}}{\partial \ln k_{i}}$ (X=S,J) and $\epsilon_{i}^{j}=\frac{\partial \ln v_{j}}{\partial \ln S_{i}}$.

Given Equations (2) and (3), we developed a strategy to investigate the role of regulation in determining the range of variation (lower/upper bounds) and state-dependency of the control coefficient $C^{*}$ of interest (here $C^{*}=C_{i}^{S,nh}$ for NADPH homeostasis and $C^{*}=C_{i}^{J,ppp}$ for flux rerouting into oxPPP) (Figure 1B). Mathematical analysis of control coefficients can be completed by performing a set of well-chosen Gaussian eliminations in Equation (3) leading to analytic functions (see the Appendix B and Appendix C for detailed examples):

(4) $$C^{*}=F\left(C^{X},S,J,r\right).$$

The general form of the function F and the asymptotic behaviors of such function for some small or large values of $r_{i}$, $S_{i}$ or $J_{i}$ provides key information about the manner how regulation impacts the control coefficient of interest (e.g., promotes NADPH homeostasis). These functions define low-dimensional manifolds in the space of control coefficients and steady-state variables. The distribution of control coefficients obtained from random sampling of model parameters p can be represented on such manifold ($C^{*}\left(p\right)=F\left(C^{S,J}\left(p\right),S\left(p\right),J\left(p\right),r\right)$) to better visualize and discriminate the regulatory and context-dependent features determining control properties.

## 3. Results

### 3.1. Distribution of Control Coefficients in Absence of Feedback Regulation

**Figure 2:**
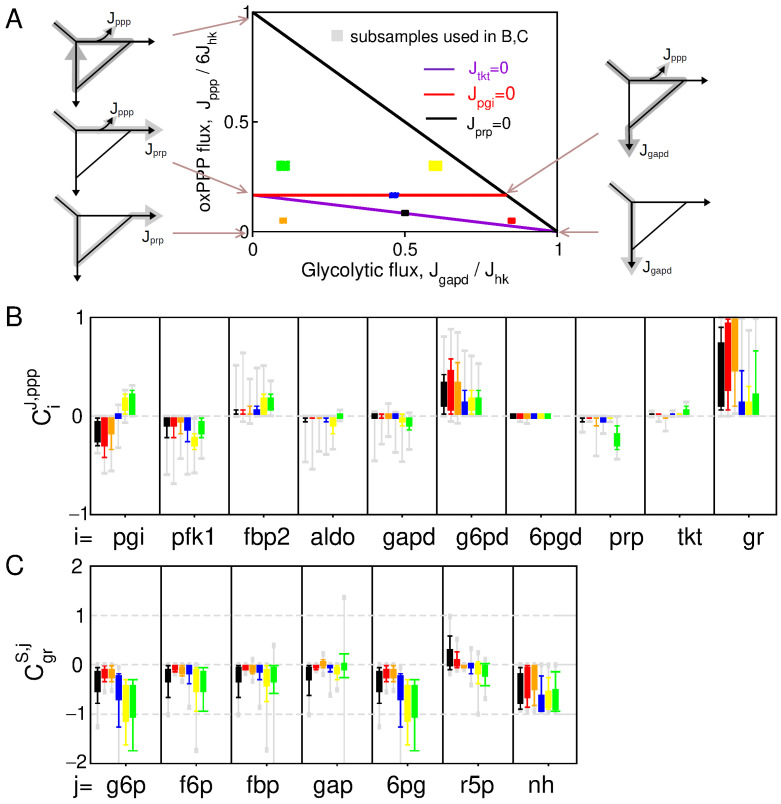
Control pattern without feedback regulation. (**A**) Partitioning of the parameter sampling procedure in different domains of the two-dimensional polytope of possible flux configurations. (**B**) Whisker-plot distribution of flux control $C_{i}^{J,ppp}$ (**B**) and concentration control $C_{gr}^{S,j}$ (**C**), obtained from parameter sampling of the 6 subdomains of the flux space (color code in (**A**)).

To provide a preliminary statistical picture of control patterns associated with NADPH homeostasis, a distribution of control coefficients can be obtained from random sampling of kinetic parameters in the absence of feedback regulations (Figure 2). This sampling approach allows us to distinguish between different contributions in the variability of control pattern: the variability due to changes in the flux state J or due to the difference in parameters associated with a given flux state J. Parameter sampling is therefore subdivided into *k* parameter subsets $p^{k}$ (size $>10^{4}$) associated with specific flux states $J^{k}$ in the space of elementary flux modes (Figure 2A). The sampled flux state is restricted to the glucose-consuming modes of the PPP, which corresponds for the kinetic model of Equation (A1) to a two-dimensional triangle polytope as function of $J_{gapd}/J_{hk}$ and $J_{g6pd}/6/J_{hk}$. In this space, the glycolytic, nucleotide-producing, NADPH-producing modes are the extremity of such polytope, and we compare control distribution in well distinct domains inside the polytope.

Parameter sampling confirms expected trends in the control pattern with many sign-definite coefficients (Figure 2B,C). NADP^+^ binding to G6PD provides a primary source of oxPPP flux increase in response to NADPH depletion where $0<C_{gr}^{J,ppp}<1$ (and $-1<C_{gr}^{S,nh}<0$) without the need of regulations. In addition, the main flux-controlling steps are G6PD, but also PFK1 and PGI, which is consistent with the notion that reduced enzyme activity in upper glycolysis leads to flux rerouting into oxPPP. However, some other features of the control pattern do not seem to match with expectations or experimental evidences. For instance, $C_{gr}^{J,ppp}$ and $C_{gr}^{S,nh}$ significantly decrease for flux state domains characterized with $J_{pgi}<0$, indicating an unlikely context-dependency of NADPH homeostasis. As well, 6PG and G6P concentration control coefficients $C_{gr}^{S,6pg/g6p}$ are strongly negative contradicting the numerous experimental evidences reporting a few-fold increase in 6PG and a moderate increase in G6P in response to oxidative stress [17,27,31]. These features suggest the involvement of regulation to enable $C_{gr}^{S,6pg/g6p}>0$ and to increase $C^{J,ppp}$ or $C^{S,nh}$ for a broader range of flux states.

### 3.2. Feedback Inhibitions of PPP and Upper Glycolysis Synergistically Cooperate for Efficient PPP Flux Rerouting

**Figure 3:**
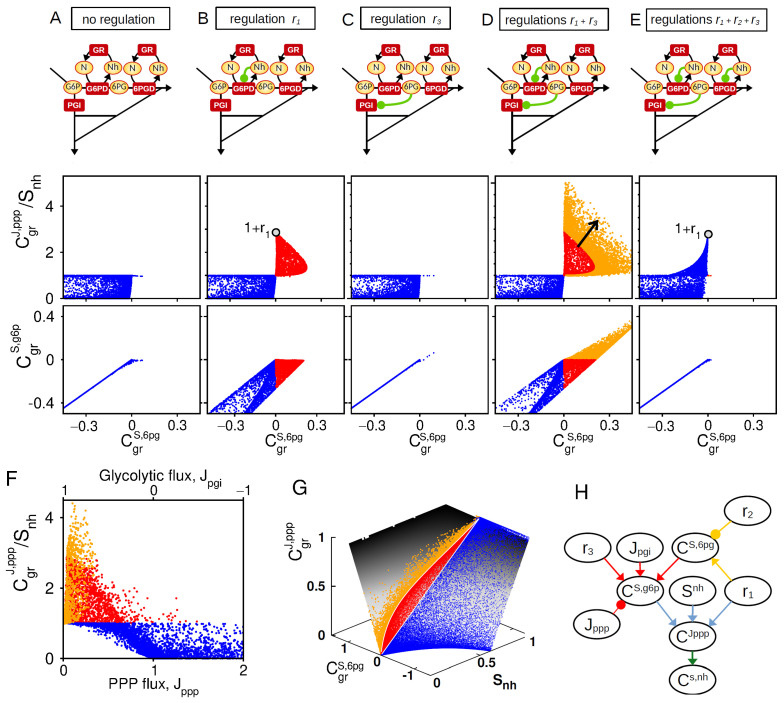
Synergistic feedback regulations for PPP flux rerouting. Comparative analysis of 5 regulatory architectures schematically represented on top: (**A**) $r_{1,2,3}=0$, (**B**) $r_{1}=2$, (**C**) $r_{3}=2$, (**D**) $r_{1,3}=2$, and (**E**) $r_{1,2,3}=2$. Middle and bottom panels represents the $C_{gr}^{J,ppp}/S_{nh}$ and $C_{gr}^{S,g6p}$ as function of $C_{gr}^{S,6pg}$, obtained from random sampling of kinetic parameters. Color code indicates different classes of behavior (Red: $C_{gr}^{S,6pg}>0$ and $C_{gr}^{S,g6p}<0$; Orange: $C_{gr}^{S,g6p}>0$, blue otherwise.) (**F**) For the architecture of panel (**D**), $C_{gr}^{J,ppp}/S_{nh}$ as function of $J_{ppp}$. (**G**) For the architecture of panel (**D**), $C_{gr}^{J,ppp}$ as function of $S_{nh}$ and $C_{gr}^{S,g6p}>0$ mapped onto the manifold related to Equation (A9). (**H**) Scheme based on Equations (5) and (A8) (colored arrows) recapitulating the interplay of $r_{1}$, $r_{2}$, and $r_{3}$ and key steady-state variables on the control associated with NADPH homeostasis.

From the matrix expression relating control and elasticity coefficients, one can derive a control equation for NADPH homeostasis as function of a set of regulatory parameters. Keeping in mind that oxPPP flux control and NADPH concentration control are related by $C_{gr}^{S,nh}=C_{gr}^{J,ppp}-1$ (Equation (A8a)), we can derive in the Appendix C.1 the following general equation:

(5) $$C_{gr}^{J,ppp}=\frac{-\epsilon_{1}J_{pgi}^{+}-\epsilon_{2}\epsilon_{3}\;J_{pgi}+J_{pgi}^{-}C_{gr}^{S,f6p}}{J_{ppp}+J_{pgi}^{+}\left(1-\epsilon_{1}\right)+J_{pgi}\epsilon_{3}\left(1-\epsilon_{2}\right)}$$

where elasticities $\epsilon_{i}$ (see Equation (A5)) depends on regulatory parameters $r_{i}$. This equation is very informative and can be analyzed in several asymptotic limits (Equation (A11)) to dissect how $C_{gr}^{J,ppp}$ depends on regulatory crosstalk, while a sampling approach exploring the parameter space of the kinetic model is required to confirm or refine the results beyond particular assumptions (Figure 3).

In the absence of regulation, an increased consumption of NADPH enhances the PPP flux control due to the concomitant increase in NADP^+^ as a cofactor of G6PD enzyme, which provides a maximum flux control of $\max\left(C_{gr}^{J,ppp}\right)=S_{nh}$ (maximum for $J_{ppp}=0$) (Equation (A11a) and Figure 3A). The regulation $r_{1}$ (NADPH-dependent inhibition of G6PD) can efficiently promote such PPP flux control to a maximum extent of $C_{gr}^{J,ppp}/S_{nh}=1+r_{1}$ for small enough $S_{nh}$ and $J_{ppp}$ (Equation (A11b) and Figure 3B). The regulation $r_{3}$ alone (6PG-dependent inhibition of PGI) does not promote PPP flux control (Equation (A11d) and Figure 3C). In sharp contrast, such allosteric regulation strongly enhances PPP flux control in presence of $r_{1}$ (Equations (A11e) and (A11f) and Figure 3D). This synergistic effect coincides with a positive control of G6P, which itself requires a strong positive control of 6PG mediated by $r_{1}$ (low panels of Figure 3B,D). The importance of a positive concentration control of 6PG and G6P is confirmed by the loss of synergistic effect for $r_{2}\geq r_{1}$ related to a loss of positive concentration control for G6P and 6PG (Figure 3E).

In the general control Equation (5), $C_{gr}^{J,ppp}$ is expected to decrease with $J_{ppp}$ while the effect of $\epsilon_{2}$ requires high glycolytic flux $J_{pgi}>0$. We therefore check that the synergistic interplay between $r_{1}$ and $r_{3}$ in promoting PPP flux control is indeed compromised for increasing (resp., decreasing) values of $J_{ppp}$ (resp., $J_{pgi}$) (Figure 3F). Finally, the key roles of $S_{nh}$ and $C^{6pg}$ can be depicted by plotting the distribution of PPP flux control of sampled model on a surface derived from Equation (A9) (Figure 3G). To summarize, the role of regulations in shaping NADPH homeostasis can be schematically represented to make apparent regulatory crosstalk and context dependencies (Figure 3H).

### 3.3. Ros-Dependent Inhibition of Glycolytic Enzymes Expands NADPH Homeostatic Abilities

After identifying a synergistic mode of allosteric regulation which is only efficient when glycolytic flux out competes oxPPP flux ($J_{pgi}>J_{ppp}$), we now examine the requirement for alternative regulatory strategies in the case where $J_{ppp}>J_{pgi}$, such as during acute oxidative stress. Indeed, an excessive, endogenous, or exogenous, production of ROS species typically leads to increased oxidation of NADPH, but also high flux rerouting where $J_{ppp}>J_{pgi}$ [18,33]. In this physiological context, H_2_O_2_, a major source of ROS, directly interacts with and inhibits several glycolytic enzymes, notably GAPD and PFKFB3, through S-gluthationylation modifications. We therefore apply now metabolic control analysis to a context where the increase in GR-dependent oxidation of NADPH into NADP^+^ is mediated by an increased production of H_2_O_2_, shifting the nature of parametric perturbation from *k_gr_* to *k_ox_* for which control coefficients are computed. The model incorporates now the H_2_O_2_-dependent oxidative inhibitions of GAPD and PFK1. In this scenario, control manifold equations can be derived (see Appendix C.2) to obtain a simple general expression for the NADPH control coefficient:

(6) $$C_{ox}^{S,nh}=\frac{J_{pgi}^{-}C_{ox}^{S,f6p}-J_{pgi}^{+}-J_{ppp}}{-\epsilon_{1}J_{pgi}^{+}}.$$

**Figure 4:**
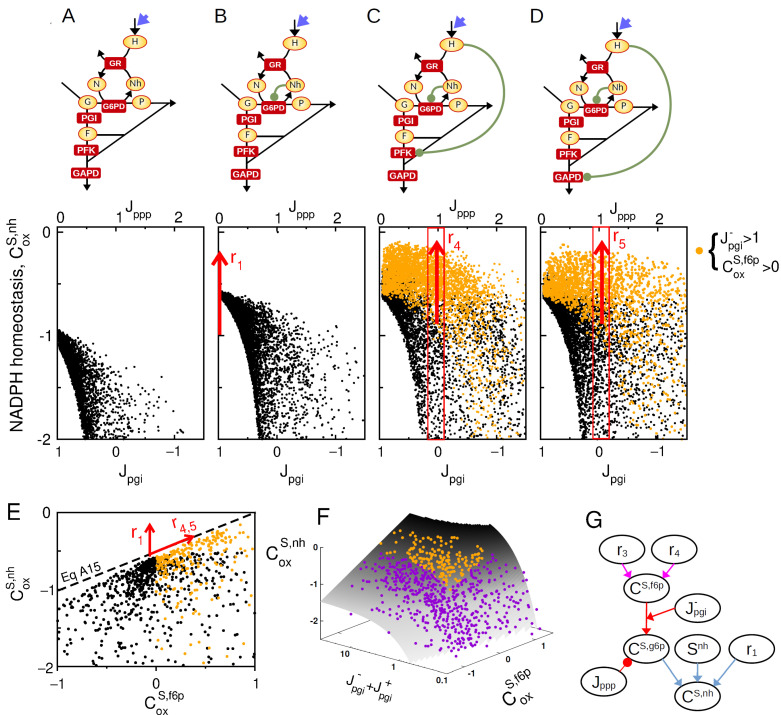
Complementary feedback regulation for NADPH homeostasis. Sampling space is restricted to the criteria $S_{nh}=S_{n}$. (**A**–**D**) $C_{ox}^{S,nh}$ as function of $J_{pgi}$ for a random sampling of kinetic models where $S_{n}=S_{nh}$. for 4 regulatory architecture ((**A**): $r_{i}=0$, (**B**): $r_{1}=5$, (**C**): $r_{1}=5$, $r_{4}=2$, (**D**): $r_{1}=5$, $r_{5}=2$). Orange colors are associated with sampled model satisfying $J_{pgi}^{-}>1$ and $C^{S,f6p}>0$. (**E**) $C_{ox}^{S,nh}$ as function $C_{ox}^{S,f6p}$ for random model sampling, highlighting the maximal bound for NADPH homeostasis. (**F**) $C_{ox}^{S,nh}$ as function $C_{ox}^{S,f6p}$ and $J_{pgi}^{-/+}$ where random model sampling are shown on the manifold obtained from Equation (6), intersected with the condition $J_{pgi}=0$. (**G**) Scheme based on Equations (6) and (A12) (colored arrows) recapitulating the interplay of $r_{1}$, $r_{4}$ and $r_{5}$ and key steady-state variables on the NADPH homeostasis control coefficient.

To analyze the control properties associated with this expression, random sampling of kinetic space is performed again to plot the statistical distribution of $C_{ox}^{S,nh}$ as function of key regulatory and steady-state parameters of Equation (6). The sampling procedure is supplemented by the criteria that $S_{nh}=S_{n}$ to leave aside the previously characterized effect of NADPH concentration. Results show that the oxidative inhibition of glycolytic enzymes by H_2_O_2_ promotes significant NADPH homeostasis, but only for negative enough level of $J_{pgi}$ (compare Figure 4A–D). Enhanced NADPH homeostasis correlates with positive values of $C_{ox}^{S,f6p}$ and high values of $J_{pgi}^{-}$ consistently with the term $J_{pgi}^{-}C_{ox}^{S,f6p}$ of Equation (6). It is to note that inhibitions of PFK1 and GAPD exhibit qualitatively the same control effect. The interplay between the feedback control of G6PD and the inhibition of lower glycolysis is depicted in Figure 4E, where the upper bound for $C_{ox}^{S,nh}$ increases with both $r_{1}$ and $C_{gr}^{S,f6p}$ in independent manner. This interplay nevertheless requires a large bidirectional flux in PGI reaction as compared to the net flux ($J_{pgi}^{+}+J_{pgi}^{-}\gg J_{pgi}$) as shown by plotting control coefficients onto the control manifold associated with Equation (6) (Figure 4F).

To summarize, the upregulation of G6PD enzyme mediated by $r_{1}$ and the inhibition of lower glycolysis mediated by $r_{4/5}$ do not display a synergistic effect, but a strong complementary effect as these two classes of regulation promote NADPH homeostasis for, respectively, $J_{ppp}$ low and high compared to $J_{pgi}$ (Figure 4G). Because these two classes of regulation are efficient for large $J_{pgi}^{-}$ and large $J_{pgi}^{+}$, respectively, their complementary effect is empowered by high bidirectional flux ($J_{pgi}^{+/-}>J_{pgi}$), supporting the need for near-equilibrium PGI reaction associated with a low Gibbs free energy $\Delta G=J_{pgi}\ln\left(J_{pgi}^{+}/J_{pgi}^{-}\right)$.

## 4. Discussion

The present study proposes a methodological framework to investigate the mode of regulatory crosstalks in the functional response of metabolic networks. The context-dependent control of metabolic fluxes involves complex regulation patterns as exemplified in the regulation of carbon flux rerouting into oxidative branch of the PPP to meet cellular demand in NADPH [16,22]. A number of experimental and computational studies has disputed the respective or prevalent roles of diverse metabolic regulations prone to contribute to such flux rerouting [17,27,31,34,35]. The present framework aims to reconcile these studies by carefully addressing context-dependency and cooperation in the regulation pattern of NADPH homeostasis. Explicit mathematical expression of control coefficients allows us to highlight to which extent a regulatory feedback may improve, or not, a metabolic function, but more importantly how such effect depends on steady-state variables and the presence of other regulations. This provides a refined picture of complex regulation patterns that disentangles several modes of regulatory interplay ranging from synergistic effects, compensatory effects or complementary effects.

Synergistic allostery usually refers to allosteric binding to separate sites of a same enzyme thereby increasing enzymatic activity in a cooperative manner [36,37]. The synergistic effect described here rather characterizes a cooperative scheme where a control coefficient associated with a given metabolic functionality is enhanced congruently by first-order effects of regulations and second-order effects where one regulation triggers concentration changes in effectors of other regulations. Such an effect falls within higher-order approaches of metabolic control analysis where, for instance, second-order control coefficients describe synergies between enzyme pairs [9,38]. The careful analysis of the cooperation between NADPH-dependent inhibition of G6PD and 6PG-dependent inhibition of PGI depicts the set of requirements for efficient synergy. For instance, increase in 6PG levels, commonly observed in response to oxidative stress [17,31], serves as a proxy to support synergistic allosteric regulation, and such an increase absolutely requires differential elasticities properties in the two main branches of the oxidative PPP controlled by G6PD and 6PGD. Specifically, 6PGD enzymes must be characterized with a lower NADPH-dependent feedback inhibition than G6PD. Although experimental measurements strongly depend on cell type and methods, comparative studies seem to indicate a larger $K_{i}$ value about ≈30 µM in 6PGD [39,40] compared to G6PD about ≈7 µM [25]. Rapid post-translation modifications upregulating specifically G6PD enzymes [41] could also contribute to differential upregulation of G6PD compared to 6PGD, required for 6PG increase and concomitant inhibition of PGI.

Control coefficients computed for a reference metabolic state can already identify a set of rate-limiting enzymes that are likely to be conjointly regulated supporting the requirement of distributed control [42,43]. In this scheme, the control of the regulator species itself is treated separately and can be metabolites or signaling hub proteins involved in metabolic regulation such as AMPK, AKT, or NRF2 [44,45,46]. However, an approach addressing how control coefficients depend on the reference metabolic state reveals more elaborate coordination between regulation, based on the notion of complementary regulatory efficiencies. In the case of carbon flux rerouting toward oxPPP, our results show that synergistic co-regulation of PGI and G6PD is the most efficient for a metabolic network operating in a pure glycolytic mode (glyclolysis flux much larger than oxPPP flux) while the inhibition of lower glycolysis is the most efficient in the case of a PPP mode or reversed glycolytic mode. The two regulation strategies can therefore relay and compensate each other in a context-dependent manner for instance related to the severity or the source of the metabolic stress. Furthermore, such complementary range of regulation efficiency requires some particular kinetic features of the metabolic network, as PGI reactions must operate close to equilibrium. This thermodynamic requirement coincides with recent experimental results that emphasize a near-equilibrium activity of PGI enzymes in various contexts [47], including oxidative bursts [48]. This latter result motivates us to refine our framework to integrate thermodynamic constraints as already implemented in some previous studies [5,7,9].

## Data Availability

The fortran scripts used in this study to perform model simulations and analysis are made available from 16 March 2023 at GitHub repository https://github.com/bpfeuty/metabolites_article.

## Abbreviations

The following abbreviations are used in this manuscript:

MCA	Metabolic control analysis
PPP	Pentose phosphate pathway
ROS	Reactive oxygen species
G6P	Glucose-6-phosphate
F6P	Fructose-6-phosphate
FBP	Fructose-1,6-bisphosphate
GAP	Glyceraldehyde-3-phosphate
6PG	6-phosphogluconate
R5P	Ribose 5-phosphate
PKM2	Pyruvate kinase muscle isozyme M2
PFKFB3	Phosphofructo-2-kinase fructose-2,6-bisphosphatase-3
NH,NADPH	Nicotinamide adenine dinucleotide phosphate hydrogen
N,NADP	Nicotinamide adenine dinucleotide phosphate
OX	Oxidative stress
GR	Glutathione reductase
HK	Hexokinase
G6PD	G6P dehydrogenase
6PGD	6PG dehydrogenase
PRP	Phosphoribosyl pyrophosphate
PGI	Phosphoglucose isomerase
PFK	Phosphofructokinase (type 1)
FBPase	Fructose-1,6-bisphosphatase
ALD	Fructose 1,6 bisphosphate aldolase
GAPD	GAP dehydrogenase
TKT	Transketolase

## Appendix A. Kinetic Model

The metabolic network of Figure 1A describes a simplified scheme of the pentose phosphate pathway comprising $n_{m}=9$ metabolite species and $n_{r}=12$ reactions. The corresponding ordinary differential equation system ds/dt=Nv reads:

(A1a) $$\begin{array}{cc} \frac{ds_{g6p}}{dt} & =v_{hk}-v_{g6pd}-v_{pgi} \end{array}$$

(A1b) $$\begin{array}{cc} \frac{ds_{6pg}}{dt} & =v_{g6pd}-v_{6pgd} \end{array}$$

(A1c) $$\begin{array}{cc} \frac{ds_{r5p}}{dt} & =v_{6pgd}-3v_{tkt}-v_{prp} \end{array}$$

(A1d) $$\begin{array}{cc} \frac{ds_{f6p}}{dt} & =2\;v_{tkt}+v_{pgi}+v_{fbp}-v_{pfk} \end{array}$$

(A1e) $$\begin{array}{cc} \frac{ds_{f6b}}{dt} & =v_{pfk}-v_{fbp}-v_{ald} \end{array}$$

(A1f) $$\begin{array}{cc} \frac{ds_{gap}}{dt} & =2\;v_{ald}+v_{tkt}-v_{gapd} \end{array}$$

(A1g) $$\begin{array}{cc} \frac{ds_{nh}}{dt} & =v_{g6pd}+v_{6pgd}-v_{gr} \end{array}$$

(A1h) $$\begin{array}{cc} \frac{ds_{n}}{dt} & =-v_{g6pd}-v_{6pgd}+v_{gr} \end{array}$$

(A1i) $$\begin{array}{cc} \frac{ds_{h2o2}}{dt} & =-v_{gr}+v_{ox}. \end{array}$$

The simplifying assumptions consist of pooling or neglecting some reactions as compared to a detailed kinetic model of PPP [18]:

- RPI, RPE, TKT1, TKT2, and TAL are pooled to form a single reaction for the nonoxidative branch of the PPP (S7P, E4P metabolites are not included).
- ALD and TPI are pooled (DHAP metabolite is not included).
- GP and GRX are pooled (GSSG, GSH are not included).
- Catalase reaction degrading H_2_O_2_ is neglected.

We consider the conservation relation $s_{n}+s_{nh}=1$, defining a concentration unit. Defining $N^{+}$ and $N^{-}$ as matrices collecting positive-valued stochiometric coefficients of the reactants and of the products, respectively, such that $N=N^{-}-N^{+}$, a general form of reaction rates $v_{i}$ reads for regulated reversible reactions:

(A2) $$v_{i}\left(\vec{s}\right)=\frac{k_{i}}{1-r_{i}s_{k}}\left(\prod_{j=1,n_{m}}s_{j}^{N_{ji}^{+}}-\prod_{j=1,n_{m}}s_{j}^{N_{ji}^{-}}/K_{i}\right)$$

where the second term is null for irreversible reactions (i.e., $K_{i}$ very large) and $s_{k}$ is the concentration of the inhibitor associated with regulation $r_{i}$. Note that $v_{hk}$ is a constant parameter. At steady-state concentration S, we have $J_{i}\equiv v_{i}\left(S\right)$ and for reversible reaction $J_{i}=J_{i}^{+}-J_{i}^{-}$ where $J_{i}^{+}$ and $J_{i}^{-}$ are the first and second terms of Equation (A2). Parameter sampling is made on the parameter $k_{i}$ and $K_{i}$ randomly chosen following a uniform distribution between $10^{-2}$ and $10^{2}$ centered around a reference value $p^{ref}={\left[1,1,0.5,1,1,2,2,0.5,0.5,1.3,0.5,2,1,2\right]}^{T}$ associated with a biologically-reasonable flux state and concentration state where:

(A3) $$p=\{k_{hk},k_{pgi},k_{pfk},k_{fbp},k_{ald},k_{gapd},k_{g6pd},k_{6pgd},k_{prp},k_{tkt},k_{gr},k_{ox},K_{pgi},K_{tkt},K_{ald}\}.$$

## Appendix B. Matrix Equation for Control Coefficients

For the metabolic network considered in the present study, the matrix relation $C^{J}=I+\epsilon \;C^{S}$ becomes a linear equation system where we focus on the control coefficients with respect to a given perturbation *x*:

(A4) $$\left(\begin{array}{c} C_{x}^{J,hk}\\ C_{x}^{J,pgi}\\ C_{x}^{J,pfk}\\ C_{x}^{J,fbp}\\ C_{x}^{J,ald}\\ C_{x}^{J,gapd}\\ C_{x}^{J,g6pd}\\ C_{x}^{J,6pgd}\\ C_{x}^{J,prp}\\ C_{x}^{J,tkt}\\ C_{x}^{J,gr}\\ C_{x}^{J,ox} \end{array}\right)=\pi_{x}+\left(\begin{array}{cccccccc} 0 & 0 & 0 & 0 & 0 & 0 & 0 & 0\\ {\tilde{J}}_{pgi}^{+} & -{\tilde{J}}_{pgi}^{-} & 0 & 0 & \epsilon_{6pg}^{pgi}\left(r_{3}\right) & 0 & 0 & 0\\ 0 & 1 & 0 & 0 & 0 & 0 & 0 & \epsilon_{h2o2}^{pfk}\left(r_{4}\right)\\ 0 & 0 & 1 & 0 & 0 & 0 & 0 & 0\\ 0 & 0 & {\tilde{J}}_{ald}^{+} & -{\tilde{J}}_{ald}^{-} & 0 & 0 & 0 & 0\\ 0 & 0 & 0 & 1 & 0 & 0 & 0 & \epsilon_{h2o2}^{gapd}\left(r_{5}\right)\\ 1 & 0 & 0 & 0 & 0 & 0 & \epsilon_{nh}^{g6pd}\left(r_{1}\right) & 0\\ 0 & 0 & 0 & 0 & 1 & 0 & \epsilon_{nh}^{6pgd}\left(r_{2}\right) & 0\\ 0 & 0 & 0 & 0 & 0 & 1 & 0 & 0\\ 0 & {\tilde{J}}_{tkt}^{+} & 0 & -{\tilde{J}}_{tkt}^{-} & 0 & {\tilde{J}}_{tkt}^{+} & 0 & 0\\ 0 & 0 & 0 & 0 & 0 & 0 & 1 & a_{x}\\ 0 & 0 & 0 & 0 & 0 & 0 & 0 & 0 \end{array}\right)\left(\begin{array}{c} C_{x}^{S,g6p}\\ C_{x}^{S,f6p}\\ C_{x}^{S,fbp}\\ C_{x}^{S,gap}\\ C_{x}^{S,6pg}\\ C_{x}^{S,r5p}\\ C_{x}^{S,nh}\\ C_{x}^{S,h2o2} \end{array}\right)$$

The perturbation corresponds either to increased NADPH consumption, (i.e., $x=k_{gr}$) or increased ROS production (i.e., $x=k_{ox}$). We use ${\tilde{J}}_{i}^{+/-}=J_{i}^{+/-}/J_{i}$ in Equation (A4) only, for notation conciseness. Elasticity coefficients are given by:

(A5a) $$\begin{array}{cc} \epsilon_{nh}^{J,g6pd}\left(r_{1}\right) & =-\frac{S_{nh}\left(1+r_{1}\right)}{\left(1-S_{nh}\right)\left(1+r_{1}S_{nh}\right)}\equiv \epsilon_{1} \end{array}$$

(A5b) $$\begin{array}{cc} \epsilon_{nh}^{J,6pgd}\left(r_{2}\right) & =-\frac{S_{nh}\left(1+r_{2}\right)}{\left(1-S_{nh}\right)\left(1+r_{2}S_{nh}\right)}\equiv \epsilon_{2} \end{array}$$

(A5c) $$\begin{array}{cc} \epsilon_{6pg}^{J,pgi}\left(r_{3}\right) & =-\frac{r_{3}\;S_{6pg}}{1+r_{3}S_{6pg}}\equiv \epsilon_{3} \end{array}$$

(A5d) $$\begin{array}{cc} \epsilon_{h2o2}^{J,pfk}\left(r_{4}\right) & =-\frac{r_{4}\;S_{h2o2}}{1+r_{4}S_{h2o2}}\equiv \epsilon_{4} \end{array}$$

(A5e) $$\begin{array}{cc} \epsilon_{h2o2}^{J,gapd}\left(r_{5}\right) & =-\frac{r_{5}\;S_{h2o2}}{1+r_{5}S_{h2o2}}\equiv \epsilon_{5}. \end{array}$$

These elasticities associated with inhibitory interactions are negative-valued (ϵ<0), with minimal value of −1 for $\epsilon_{3,4,5}$. The calculations in Appendix C may use the asymptotic property that $\lim_{S_{nh}\to 0}\epsilon_{1,2}=S_{nh}\left(1+r_{1,2}\right)$.

For a given set of parameters $p^{*}$, the steady state equation $N\;v(S\left(p^{*}\right),p^{*})=0$ has a unique solution $\left\{S^{*},J^{*}\right\}=f\left(p^{*}\right)$. Associated to a particular steady-state, the above equation thus gives a unique solution for control coefficient $C^{J,S}=f\left(p^{*}\right)$ which are computed during the parameter sampling procedure.

## Appendix C. Computation of Control Manifolds

### Appendix C.1. Control Analysis of Regulatory Crosstalk r_*1,2,3*_

A first setting of the problem focuses on the interplay between the regulation of oxPPP enzymes and PGI ($r_{1,2,3}$ in Figure 3). We consider a perturbation of the oxidation rate of NADPH where x=gr, $\pi_{gr}={\left[0,0,0,0,0,0,0,0,0,1,0\right]}^{T}$ and $a_{gr}=0$ in Equation (A4). We define $J_{ppp}$ as the steady-state flux through the oxidative branch of the PPP, which relates to some other steady-state fluxes as:

(A6) $$J_{ppp}\equiv J_{g6pd}=J_{6pgd}=J_{gr}/2=J_{hk}-J_{pgi}\;.$$

These equalities and the condition that $C_{gr}^{J,hk}=0$ (first equation of Equation (A4)) translate into:

(A7) $$C_{gr}^{J,ppp}\equiv C_{gr}^{J,g6pd}=C_{gr}^{J,6pgd}=C_{gr}^{J,gr}=-\frac{J_{pgi}}{J_{ppp}}\;C_{gr}^{J,pgi}\;.$$

We select a subset of equation associated with the system of Equation (A4) which involves the regulation-dependent elasticity coefficients $\epsilon_{i=1,2,3}$ in order to obtain 4 control equations for $C_{gr}^{J,ppp}$

(A8a) $$\begin{array}{cc} C_{gr}^{J,ppp} & =1+C_{gr}^{S,nh} \end{array}$$

(A8b) $$\begin{array}{cc} C_{gr}^{J,ppp} & =\epsilon_{1}C_{gr}^{S,nh}+C_{gr}^{S,g6p} \end{array}$$

(A8c) $$\begin{array}{cc} C_{gr}^{J,ppp} & =\epsilon_{2}C_{gr}^{S,nh}+C_{gr}^{S,6pg} \end{array}$$

(A8d) $$\begin{array}{cc} C_{gr}^{J,ppp} & =\left(-J_{pgi}^{+}C_{gr}^{S,g6p}+J_{pgi}^{-}C_{gr}^{S,f6p}-\epsilon_{3}C_{gr}^{S,6pg}J_{pgi}\right)/J_{ppp}\;. \end{array}$$

Combining Equations (A8a) and (A8b) yields:

(A9) $$C_{gr}^{J,ppp}=\frac{-\epsilon_{1}+C_{gr}^{S,g6p}}{1-\epsilon_{1}}\;.$$

Combining Equation (A8), a more general control expression is obtained:

(A10) $$C_{gr}^{J,ppp}=\frac{-\epsilon_{1}J_{pgi}^{+}-\epsilon_{2}\epsilon_{3}\;J_{pgi}+J_{pgi}^{-}C_{gr}^{S,f6p}}{J_{ppp}+J_{pgi}^{+}\left(1-\epsilon_{1}\right)+J_{pgi}\epsilon_{3}\left(1-\epsilon_{2}\right)}\;.$$

Assuming a pure glycolytic mode $J_{pgi}=J_{pgi}^{+}=J_{hk}$, we obtain a set of asymptotic relations:

(A11a) $$\begin{array}{c} \max C_{gr}^{J,ppp}\left(r_{i}=0\right)=S_{nh} \end{array}$$

(A11b) $$\begin{array}{c} \lim_{S_{nh}\to 0}C_{gr}^{J,ppp}\left(r_{3}=0\right)=S_{nh}\left(1+r_{1}\right) \end{array}$$

(A11c) $$\begin{array}{c} \lim_{S_{nh}\to 0}C_{gr}^{J,ppp}=S_{nh}\left(\frac{1+r_{1}+\epsilon_{3}\left(1+r_{2}\right)}{1+\epsilon_{3}}\right) \end{array}$$

(A11d) $$\begin{array}{c} \lim_{S_{nh}\to 0}C_{gr}^{J,ppp}\left(r_{1,2}=0\right)=S_{nh}<1 \end{array}$$

(A11e) $$\begin{array}{c} \lim_{S_{nh}\to 0}C_{gr}^{J,ppp}\left(r_{2}=0,,r_{3}\ll 1\right)=S_{nh}\left(1+r_{1}+r_{1}r_{3}S_{6pg}\right) \end{array}$$

(A11f) $$\begin{array}{c} \lim_{S_{nh}\to 0}C_{gr}^{J,ppp}\left(r_{2}=0,r_{3}\gg 1\right)=S_{nh}r_{1}r_{3} \end{array}$$

- All those asymptotic relations are proportional to $S_{nh}$, justifying the use of the normalized control coefficient $C_{gr}^{J,ppp}/S_{nh}$ (Figure 3).
- Equation (A11a) shows that a PPP flux control driven by NADPH_+_ cofactor binding to G6PD (no regulation) has an upper bound of $S_{nh}$.
- Equation (A11b) expresses that $r_{1}$ promotes PPP flux control (i) independently on $r_{2}$, (ii) especially for small $S_{nh}$.
- Equation (A11c) defines the complex nonlinear interplay between contributions of $r_{1}$, $r_{2}$, and $r_{3}$.
- Equation (A11d) shows indeed that $r_{3}$ alone, even very large, cannot increase PPP flux control.
- Equations (A11e) and (A11f) indicate a synergistic cooperation between $r_{1}$ and $r_{3}$ (product term) for both small or large values of $r_{3}$.

Beyond the assumption of a pure glycolytic mode, Equation (A10) contains the terms $J_{ppp}$ in the denominator and $J_{pgi}$ in factor of $\epsilon_{3}$, which means that flux control decreases with increasing value of $J_{ppp}$ and that the effect of $\epsilon_{2}$ cancels for $J_{pgi}=0$ (i.e., pure oxidative PPP mode).

### Appendix C.2. Control Analysis of Regulatory Crosstalk r 1,4,5

An alternative setting of the problem focuses on the interplay between the regulations of oxPPP enzymes and PFK1 or GAPD ($r_{1,4,5}$ in Figure 4). The perturbation of NADPH homeostasis is now due to an increased production rate of H_2_O_2_ or ROS (i.e., $k_{ox}$) where $\pi_{ox}={\left[0,0,0,0,0,0,0,0,0,0,1\right]}^{T}$ and $a_{ox}=1$ in Equation (A4). For such perturbation scheme, the relevant set of control equations becomes:

(A12a) $$\begin{array}{cc} C_{ox}^{J,ppp} & =1 \end{array}$$

(A12b) $$\begin{array}{cc} C_{ox}^{J,ppp} & =\epsilon_{1}\;C_{ox}^{S,nh}+C_{ox}^{S,g6p} \end{array}$$

(A12c) $$\begin{array}{cc} C_{ox}^{J,ppp} & =\epsilon_{2}C_{ox}^{S,nh}+C_{ox}^{S,6pg} \end{array}$$

(A12d) $$\begin{array}{cc} C_{ox}^{J,ppp} & =\left(-J_{pgi}^{+}C_{ox}^{S,g6p}+J_{pgi}^{-}C_{ox}^{S,f6p}-\epsilon_{3}C_{gr}^{S,6pg}J_{pgi}\right)/J_{ppp} \end{array}$$

(A12e) $$\begin{array}{cc} C_{ox}^{J,ppp} & =C_{ox}^{S,nh}+C_{ox}^{S,h2o2} \end{array}$$

(A12f) $$\begin{array}{cc} C_{ox}^{J,pfk} & =C_{ox}^{S,f6p}+\epsilon_{4}C_{ox}^{S,h2o2}, \end{array}$$

where one obtains after combining Equations (A12a)–(A12d):

(A13) $$C_{ox}^{S,nh}=\frac{J_{ppp}+J_{pgi}^{+}+\epsilon_{3}J_{pgi}-J_{pgi}^{-}C_{ox}^{S,f6p}}{J_{pgi}^{+}\epsilon_{1}+\epsilon_{3}\epsilon_{2}J_{pgi}}\;,$$

which finally simplifies for $\epsilon_{3}=0$ to:

(A14) $$C_{ox}^{S,nh}=\frac{J_{pgi}^{-}C_{ox}^{S,f6p}-J_{pgi}^{+}-J_{ppp}}{-\epsilon_{1}J_{pgi}^{+}}\;.$$

NADPH homeostasis is related to smallest $|C_{ox}^{S,nh}|$ values as possible and can be promoted by several mechanisms:

- Positive values of $C_{ox}^{S,f6p}$ which is increased by regulation $r_{4}$ following Equation (A12f), but also regulation $r_{5}$.
- The effect of those regulations is enhanced by large directional PGI flux $J_{pgi}^{-}$ from F6P to G6P.
- The effect of $r_{1}$ is amplified by large directional PGI flux $J_{pgi}^{+}$ from G6P to F6P.
- The two items above indicate that efficient regulation of NADPH homeostasis by $r_{1,4,5}$ requires high values of both $J_{pgi}^{+,-}$ with an upper bound given by:
  (A15) $$C_{ox}^{s,nh}=\frac{C_{ox}^{S,f6p}-1}{-\epsilon_{1}}.$$
